# RhoA/ROCK Signaling Regulates Drp1-Mediated Mitochondrial Fission During Collective Cell Migration

**DOI:** 10.3389/fcell.2022.882581

**Published:** 2022-05-31

**Authors:** Chen Qu, Wen Yang, Yating Kan, Hui Zuo, Mengqi Wu, Qing Zhang, Heng Wang, Dou Wang, Jiong Chen

**Affiliations:** ^1^ MOE Key Laboratory of Model Animals for Disease Study, Model Animal Research Center, Medical School of Nanjing University, Nanjing, China; ^2^ Jiangsu Key Laboratory of Molecular Medicine, Medical School of Nanjing University, Nanjing, China; ^3^ TEDA Institute of Biological Sciences and Biotechnology, Nankai University, Tianjin, China; ^4^ State Key Laboratory of Molecular Developmental Biology, Institute of Genetics and Developmental Biology, Chinese Academy of Sciences, Beijing, China

**Keywords:** collective migration, DRP1, RhoA/ROCK signaling, Drosophila border cells, mitochondrial dynamics, actomyosin dynamics

## Abstract

Collective migration plays critical roles in developmental, physiological and pathological processes, and requires a dynamic actomyosin network for cell shape change, cell adhesion and cell-cell communication. The dynamic network of mitochondria in individual cells is regulated by mitochondrial fission and fusion, and is required for cellular processes including cell metabolism, apoptosis and cell division. But whether mitochondrial dynamics interplays with and regulates actomyosin dynamics during collective migration is not clear. Here, we demonstrate that proper regulation of mitochondrial dynamics is critical for collective migration of *Drosophila* border cells during oogenesis, and misregulation of fission or fusion results in reduction of ATP levels. Specifically, *Drp1* is genetically required for border cell migration, and Drp1-mediated mitochondrial fission promotes formation of leading protrusion, likely through its regulation of ATP levels. Reduction of ATP levels by drug treatment also affects protrusion formation as well as actomyosin dynamics. Importantly, we find that RhoA/ROCK signaling, which is essential for actin and myosin dynamics during border cell migration, could exert its effect on mitochondrial fission through regulating Drp1’s recruitment to mitochondria. These findings suggest that RhoA/ROCK signaling may couple or coordinate actomyosin dynamics with mitochondrial dynamics to achieve optimal actomyosin function, leading to protrusive and migratory behavior.

## Introduction

Cell migration is critical for such developmental, physiological and pathological processes as tissue and organ formation, wound healing and tumor metastasis. Cells not only migrate individually but can also migrate collectively as a coherent group (Friedl and Gilmour, 2009; Rørth, 2011). A common feature of both forms of cell migration is that migratory cells undergo constant shape change as mediated by the dynamic actomyosin network. Actin dynamics arise from the ATP-dependent process of actin treadmilling, which consists of the polymerization of ATP-bound actin monomer (ATP-G-actin) onto the plus (barbed) end of the actin filament (F-actin), hydrolysis of ATP within the incorporated actin subunits, depolymerization of ADP-G-actin from the minus (pointed) end, and exchange of ATP for ADP resulting in ATP-G-actin being used for another round of polymerization onto the plus end (Chen et al., 2000). The dynamics of non-muscle Myosin II stems from the fact that Myosin II could assemble onto or dissemble from the F-actin, and the ATP-dependent motor activity of Myosin II could be regulated by phosphorylation of its regulatory light chain or by the availability of ATP (Garrido-Casado et al., 2021). A variety of actin- and Myosin II- interacting proteins have been studied for their roles in regulating the dynamics of actomyosin network, but whether and how ATP generation affects the dynamics and function of actomyosin network and how ATP generation is regulated during cell migration are much less understood. Signaling from the Rho family GTPases including Rac, Cdc42 and Rho regulate different aspects of actomyosin function during cell migration, resulting in formation of lamellipodial protrusions, filopodia, actomyosin stress fibers respectively (Raftopoulou and Hall, 2004). But whether these signaling pathways cross talk with ATP-regulatory cellular processes to regulate these dynamic structures is not known.

Mitochondria, the major ATP-producing organelles, also exist as a dynamic network, which can be remodeled by fission and fusion to change size and distribution within cells (Chan et al., 2012; Hoppins, 2014). Mitochondria dynamics result from two opposing processes: mitochondrial fission and fusion, which are regulated by the members of highly conserved dynamin related proteins (DRPs) family. The mitochondrial fission protein, Drp1, mediates mitochondrial outer membrane scission, whereas mitofusins (Mfn) 1 and 2, also DRP family members, carry out outer membrane fusion. Because of their importance, these proteins are regulated at the transcription, translation and post-translation modification levels. Mitochondrial dynamics have been shown to play important roles in a variety cellular processes, including cell metabolism, cell division, and cell differentiation (Hoppins, 2014). However, whether mitochondrial dynamics plays essential roles in cell migration, especially collective cell migration, through regulation of ATP generation, is not well understood.

Border cell migration in the *Drosophila* ovary is an established model system for studying collective cell migration (Friedl and Gilmour, 2009; Rørth, 2011; Montell et al., 2012). Beginning at early stage 9 of *Drosophila* oogenesis, six outer migratory border cells and two central non-migratory polar cells form a coherent cluster, detach from the epithelial layer of anterior follicle cells, invade the underlying germline tissue of nurse cells, and migrate posteriorly between the large nurse cells. By late stage 9 or early stage 10 (about 6 h later), the border cell cluster would have migrated collectively about 150 μm and reached the oocyte border between nurse cells and oocyte, hence the name border cells (Montell et al., 2012). Because of the genetic tractability and the ease of live imaging, border cells have been used extensively for studying the effects of actin and myosin dynamics on collective migration (Montell, 2003; Montell et al., 2012). Specifically, studies from our and other laboratories have demonstrated that Rac signaling promotes the formation of the predominant leading protrusion, which requires both actin polymerization mediated by Arp2/3 complex and actin depolymerization mediated by cofilin (Duchek et al., 2001; Wang et al., 2010; Zhang et al., 2011; Wang et al., 2018; Wang et al., 2020). Rho and its downstream effector ROCK have also been shown to be required for border cell migration and ROCK’s effects on Myosin II phosphorylation likely play roles in detachment of border cells from follicle epithelium, cluster cohesion, and protrusion formation (Murphy and Montell, 1996; Llense and Martín-Blanco, 2008; Zhang et al., 2011; Majumder et al., 2012). Recently, we have demonstrated that a dynamic supracellular actomyosin network at the peripheral surface of border cell cluster mediates cell-cell communication and acts to restrict the predominant protrusion to the front of cluster (Wang et al., 2020). In addition, Rho regulates the formation of the supracellular actomyosin network through ROCK (Wang et al., 2020).

In this study, we demonstrate that Drp1 is required for border cell migration and Drp1-mediated mitochondrial fission plays important roles in promoting leading protrusion and optimal ATP levels, which are essential for actin and myosin dynamics in migratory border cells. Furthermore, signaling from Rho and ROCK promotes mitochondrial fission through regulating Drp1’s recruitment to mitochondria. These findings suggest that RhoA and ROCK may couple or coordinate the dynamics of actomyosin network with the dynamics of mitochondrial network to achieve optimal actin and myosin function, leading to protrusive and migratory behavior.

## Methods

### *Drosophila* Genetics

Flies were cultured following standard procedures at 25°C except for RNAi experiments at 29°C. All strains were obtained from the Bloomington *Drosophila* Stock Center (BDSC), National Institute of Genetics Stock Center (NIG), and Vienna *Drosophila* RNAi Center (VDRC). Stocks used were: *UAS-mito-GFP* (8443), *UAS-Lifeact-GFP* (35544), *UAS-RhoA V14* (7330), *UAS-ROCK.CAT* (6667), *UAS-Mfn RNAi* (55189 and 31157), *UAS-Mfn* (67157), *UAS-Drp1* (51647), *Sqh-GFP* (57144), *Drp1* ^*1*^ (24885) and *Drp1* ^*2*^ (24899) from BDSC, *UAS-Drp1 RNAi* (3210R-3 and 3210R-4) from NIG, *UAS-Mfn RNAi* (40478) from VDRC. Mutant FRT clones were induced using hs-FLP. Flies were heat shocked for 1 h per day at 37°C for 3 days before eclosion. The newly eclosed females were raised on fresh food with yeast paste for 3 days prior to dissection.

### Immunostaining

Fly ovaries were dissected in phosphate-buffered saline (PBS), and then fixed in 4% formaldehyde for 10 min. After washes in PBST (PBS with 0.3% Triton X-100), ovaries were incubated with blocking solution (PBST containing 10% goat serum) for 30 min and then stained overnight at 4°C. The primary antibodies used were mouse anti-ATP5A (ab14748). After overnight staining, ovaries were incubated with secondary antibodies (1:200, Jackson) for 2 h at room temperature. For S2 cell immunostaining, 48 h after transfection, cells were harvested and washed with PBS. Cells were fixed in 4% formaldehyde in PBS buffer for 20 min at room temperature, treated with PBST for 20 min, and washed with PBS for 20 min three times. Mitotracker was used to label mitochondria. F-actin was labeled by Rhodamine-phalloidin (P1951, Sigma). Image of cells were acquired by Zeiss 880 confocal microscope (with Airyscan).

### Live Imaging and Drug Treatments

Egg chambers were dissected from ovaries for live imaging as described previously (Bianco et al., 2007). Egg chambers were dissected in live imaging medium and then transferred to an 8 chamber (No.155411, Thermo Fisher, United States), with each chamber containing 200 μl medium. For drug treatment, egg chambers were continuously imaged for 20–30 min by Zeiss 880 confocal microscope, before adding Oligomycin (5, 50 and 500 nM) or DMSO to the media and further live imaging.

### Measurement of Mitochondrial Morphology

For S2 cells, mitochondria were stained with Mitotracker for 30 min to detect their morphologies. Mitochondrial morphology was analyzed with Mitochondria Analyzer (Chaudhry et al., 2020), a plugin of Image J software widely used for characterizing mitochondrial morphology in cultured cells (Mao et al., 2021; Stifel et al., 2022).

### Cell Culture and Transfection

S2 cells were maintained at 25°C in Schneider’s *Drosophila* medium (S9895, Sigma) supplemented with 10% FBS (F0718, Gibco), 100 U/ml penicillin (Life Technologies), and 100 ug/ml streptomycin. *Drosophila* S2 cells were transfected using PEI (Polyethylenimine, 1 μg/μl) with final DNA concentration of 1 μg/ml and final PEI concentration of 3 μg/ml in total culture media. Drp1-HA, Mfn-HA, RhoA-HA, ROCK-HA and ROCK-Fg were subcloned into pUAST-3xFg or pUAST-3xHA vectors and used for transfection of S2 cells.

### Western Blot and Immunoprecipitation

The western blot and Immunoprecipitation experiments were performed as previously described (Kang et al., 2018; Qu et al., 2019). Briefly, cell lysates were incubated with anti-HA antibody (Santa Cruz) overnight at 4°C with end-over-end rotation. The samples were further incubated with protein A/G agarose beads (Santa Cruz) for 1 h and rinsed twice with IP wash buffer. The beads were resuspended in ×2 loading buffer for 10 min at 95°C before loading onto SDS-PAGE gels and immunoblotting. The following antibodies were used for immunoprecipitation and western blot: mouse anti-HA (1:2000); mouse anti-Fg (1:3000); mouse anti-Actin (1:5000) and goat anti-mouse HRP (1:10000). The blot was visualized using a chemiluminescent detection kit.

### Mitochondrial Isolation and ATP Assay

The mitochondria were isolated using the Mitochondria Isolation Kit (Best Bio, Shanghai, China) for ovaries and S2 cells, according to the manufacturer’s protocol. ATP concentrations were measured with enhanced ATP assay kit (Beyotime, Shanghai, China) according to the manufacturer’s protocol. Luminescence was measured on an Infinite M200Pro multifunction reader. The relative ATP levels were calculated by dividing the luminescence by total protein concentration, which was determined by Bradford method**.**

### Transmission Electron Microscopy

For TEM sample preparation, adult ovaries were dissected in PBS, and then fixed in 2.5% Glutaraldehyde for more than 48 h at 4°C. Samples were then washed three times with phosphate buffer (PB, 0.1M Na_2_HPO_4_ and 0.1M NaH_2_PO_4_, PH7.4), and post-fixed with 1% osmium tetroxide for about 2 h at 4°C. Samples were dehydrated with 2% uranyl acetate buffer overnight at 4°C, and then gradually dehydrated through graded ethanols (30%, 50%, 70%, 90%, and 100%, respectively). Samples were finally dehydrated in acetone for two times with 15 min each time, before being embedded in Spurr low viscosity embedding agent resin (SPI, United States) and cured in 70°C for 24 h. Ultrathin sections were cut on a Leica microtome and imaging was performed using a HITACHI HT7800 TEM.

## Results

### Drp1-Mediated Fission is Required for Border Cell Migration

**FIGURE 1:**
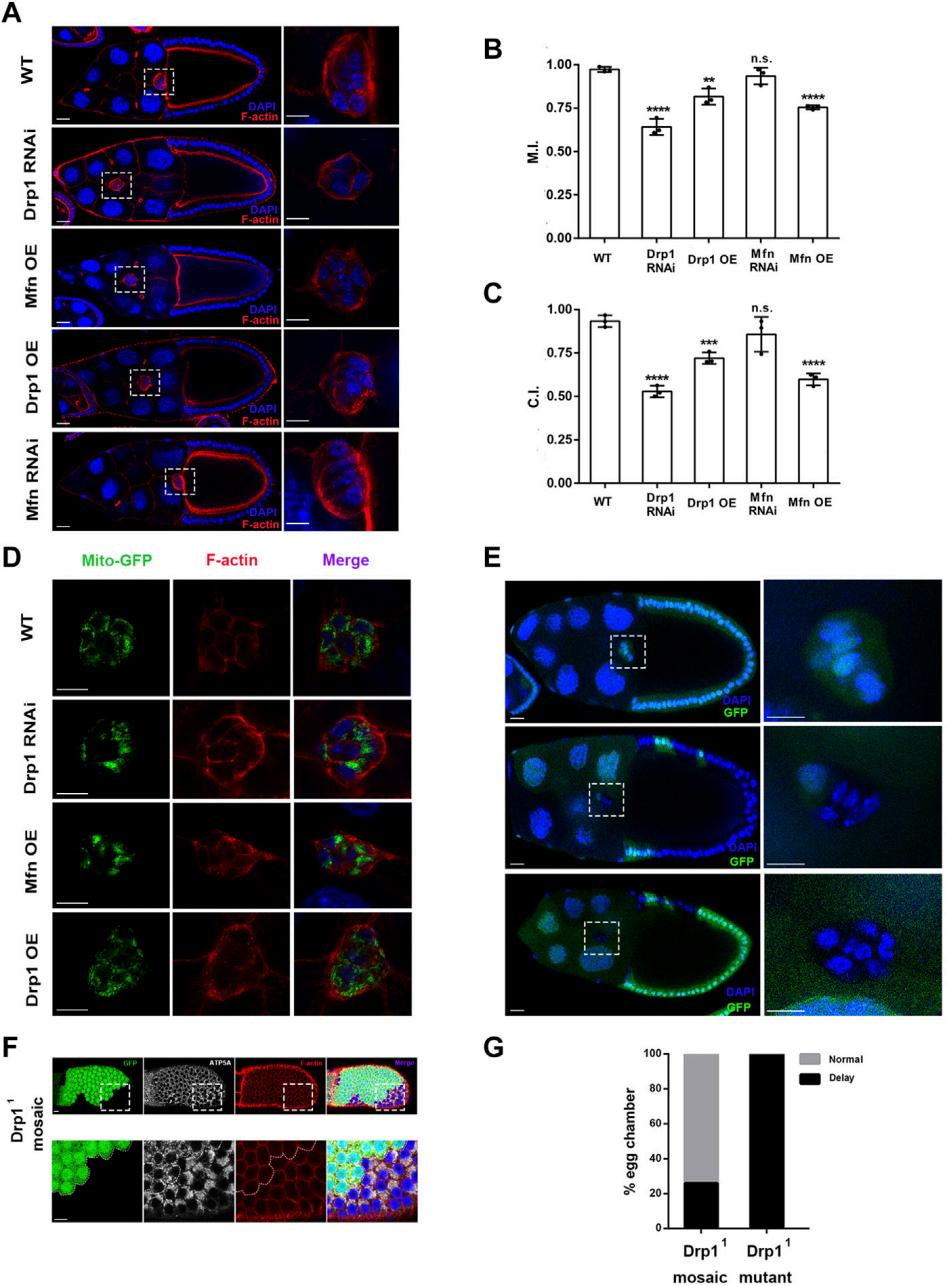
Drp1-mediated mitochondrial fission is required for border cell migration. **(A)** Confocal images of stage 10 egg chambers stained with phalloidin (for F-actin) and DAPI (for nuclei) with indicated genotypes. Border cell clusters are highlighted by the boxed regions, which are enlarged and shown to the right. Border cells expressing *Drp1 RNAi*, *Drp1* (i.e., *Drp1* overexpression, *Drp1 OE*) and *Mfn* (*Mfn OE*) by *slbo-Gal4* failed to reach the oocyte border at stage 10 of oogenesis, whereas wildtype (WT) and *Mfn RNAi* expressing border cell cluster of stage 10 egg chamber has reached the oocyte border. **(B)** The migration index (M.I.) of border cells with *Drp1 RNAi*, *Drp1 OE* and *Mfn OE* was significantly reduced as compared to the control, whereas the M.I. of *Mfn RNAi* did not show significant difference from the WT control. M.I. quantifies the migratory ability of border cells and has been described previously (Wang et al., 2018), and M.I. is calculated as followed: M.I. = [1 × n (100%) + 0.75 × n (75%) + 0.5 × n (50%) + 0.5 × n (25%) + 0 × n (0%)]/n (total). n (total) represents the total number of stage 10 egg chambers examined for each experiment. Out of the total number, n (100%) represents the number of stage 10 egg chambers in which border cells have completed or migrated 100% of the migratory route, whereas n (0%), n (25%)… represents number of stage 10 egg chambers where border cells have migrated 0%, 25% … of the migratory route respectively. **(C)** The completion index (C.I.) of border cells with *Drp1 RNAi*, *Drp1 OE* and *Mfn OE* was significantly decreased as compared to the control, whereas the C.I. of *Mfn RNAi*, did not show significant difference from the control. C.I. represents another way to quantify the border cell migration and has been described previously (Combedazou et al., 2017), and it is calculated as followed: C.I. = n (100%)/n (total). n (total) represents the total number of stage 10 egg chambers examined for each experiment, and n (100%) represents the number of stage 10 egg chambers where border cells have completed 100% of the migratory route. Three experiments have been repeated for each genotype (thus three data points for each genotype), and a total of 286, 186, 178, 165, and 335 egg chambers have been examined for WT, *Drp1 RNAi*, *Drp1 OE*, *Mfn RNAi*, and *Mfn OE* respectively for the M.I. or C.I. determination. **(D)** Single confocal sections of egg chambers stained with Mito-GFP (for mitochondria), phalloidin and DAPI with indicated genotypes. High resolution images were acquired with Zeiss 880, using Airyscan setting (near super-resolution). Patches of strong GFP fluorescence in *Drp1 RNAi* and *Mfn OE* indicate increase of mitochondrial fusion as compared to the control. **(E)** Images of stage 10 egg chambers with border cell clusters composed of all heterozygous cells (considered as a wildtype control, top panel), homozygous mutant *Drp1* ^*1*^ cells and one heterozygous cell (middle panel), and all mutant *Drp1* ^*1*^ border cells (bottom panel). Wildtype border cells reach the oocyte border by stage10, whereas mosaic and mutant border cell clusters failed to reach the border. The boxed regions indicate border cell clusters and are enlarged and shown to the right. Yellow dotted lines label the mutant clones of *Drp1* ^*1*^ border cells. **(F)** Images of mosaic *Drp1* ^*1*^ follicle epithelia stained with antibody against ATP5A (a subunit of mitochondrial ATP synthase, labeling mitochondria). The *Drp1* ^*1*^ mutant clone was marked by the absence of GFP. **(G)** 26% of stage 10 egg chambers containing *Drp1* ^*1*^ mosaic border cell clusters displayed migration delay, while 100% of egg chambers containing only *Drp1* ^*1*^ border cells displayed migration delay. Scale bars: 10 μm for border cells and follicle cells, 20 μm for egg chamber. Error bars indicate SD. Student’s *t*-test was used to determine statistical significance in this and all subsequent experiments. **p < 0.01, ***p < 0.001, ****p < 0.0001, n.s., not significant.

In order to determine the roles of mitochondrial dynamics in border cell migration, we first examined whether Drp1 and Mfn are essential for border cell migration. Two to three different RNAi lines for *Drp1* or *Mfn* were used to confirm their phenotypes. *slbo-Gal4*, a border cell-specific Gal4 driver was used to drive expression of various RNAi transgenes in border cells. *Drp1 RNAi* and *Mfn* overexpression both resulted in phenotype of strong migration delay as compared with the wild-type control (Figures 1A–C). The migration index (M.I.) of the control was 0.97 ± 0.01 (maximum value being 1), whereas those of *Drp1 RNAi* and *Mfn* overexpression were 0.64 ± 0.03 and 0.75 ± 0.01, respectively, indicating strong migration defects. The completion index (C.I.) of the control was 0.94 ± 0.02 (maximum value being 1), whereas those of *Drp1 RNAi* and *Mfn* overexpression were 0.53 ± 0.02 and 0.59 ± 0.02, respectively. The M.I. and C.I. of *Drp1* overexpression was 0.82 ± 0.03 and 0.72 ± 0.02, respectively, displaying moderate migration defects (Figures 1A–C). However, there was no significant difference between M.I. and C.I. of *Mfn RNAi* as compared with the wild-type control (Figures 1A–C). Together, these results indicate that *Drp1* but not *Mfn* is genetically required for border cell migration. Consistently, border cell clusters containing homozygous *Drp1* ^*1*^ or *Drp1* ^*2*^ mutant clones also caused migration defects (Figure 1E and data not shown). Specifically, 100% of clusters containing only *Drp* ^*1*^ mutant border cells displayed migration delay (*n* = 15), while 26% of mosaic clusters containing both wildtype and mutant border cells displayed migration delay (*n* = 19) (Figure 1G).

Expression of Mito-GFP, a widely used mitochondrial marker (Chen, 2020), in border cells revealed that both *Drp1 RNAi* and *Mfn overexpression (OE)* resulted in a morphology of increased mitochondrial fusion as compared with the wildtype control (Figure 1D). The *Mfn OE* or *Drp1 RNAi* border cell cluster contains a number of large patches of bright Mito-GFP signals in the focal plane that reflect a highly fused or clustered mitochondrial network (with *Mfn OE* more severe than *Drp1 RNAi*), whereas the control or *Drp1 OE* border cell cluster contains mostly scattered small-sized dots of Mito-GFP signals that indicate a more fragmented mitochondrial network. In addition, likely due to their small size, mitochondria in individual border cells from the control or *Drp1 OE* are much more uniformly distributed around nucleus and throughout cytoplasm than those in the *Mfn OE* or *Drp1 RNAi* border cells. In contrary, the large patches of Mito-GFP in the *Mfn OE* or *Drp1 RNAi* border cells are often asymmetrically distributed or concentrated in a corner of individual cell (i.e., on one side of the nucleus and not around nucleus). This phenomenon is likely resulted from most mitochondria within a cell organizing themselves into one or two highly-fused networks, whose large size prevent them from distributing evenly throughout the cytoplasm of the cell. Similarly, clones of *Drp1* ^*1*^ mutant follicle cells also resulted in increased mitochondrial fusion as compared with the control (Figure 1F). Taken together, these data indicate that Drp1-mediated mitochondrial fission is required for border cell migration. In comparison, Mfn-mediated mitochondrial fusion does not seem to be as critical during border cell migration.

### Drp1-Mediated Fission Promotes Leading Protrusion of Border Cells and Regulates ATP Levels

**FIGURE 2:**
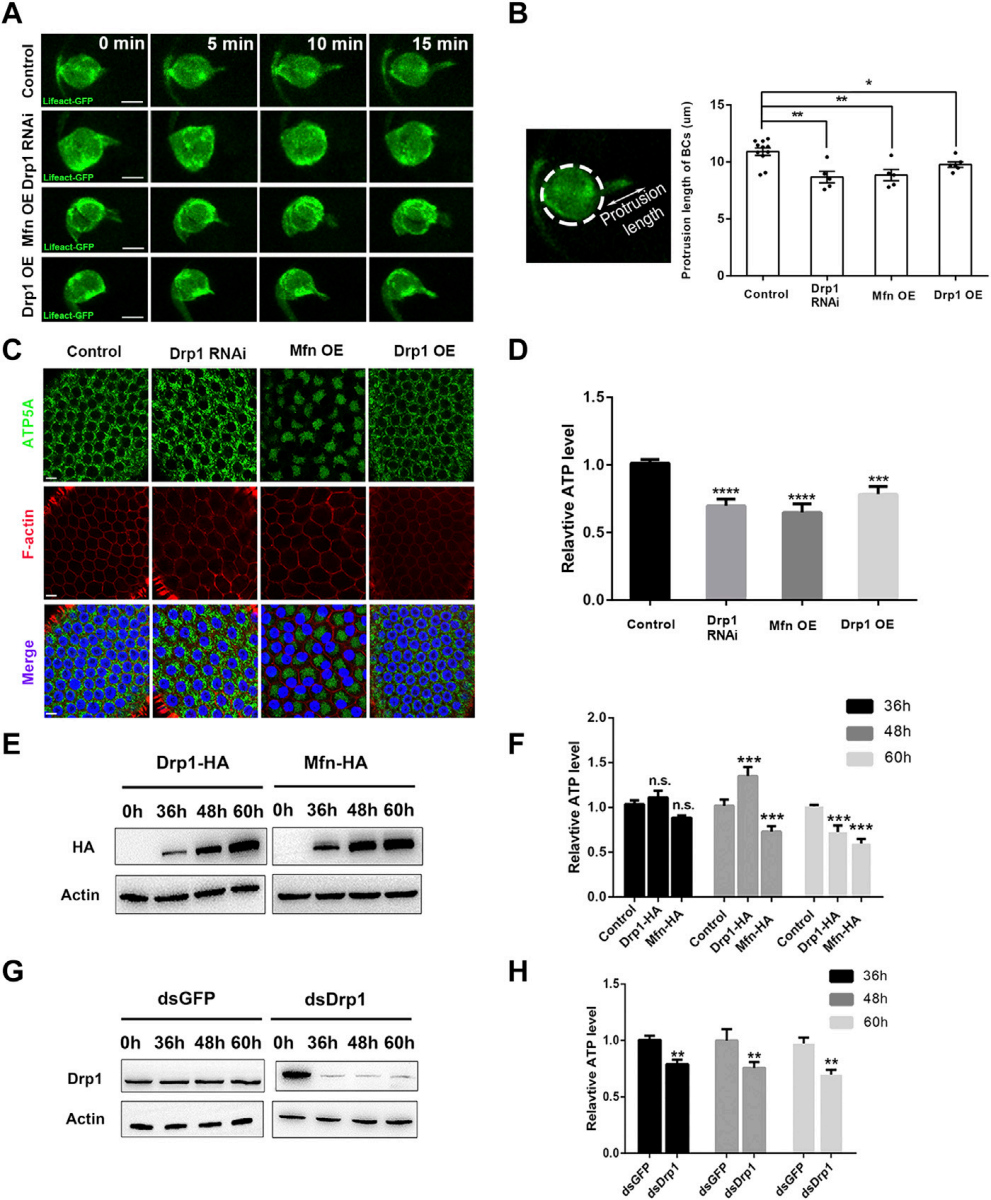
Drp1-mediated fission promotes leading protrusion through its regulation of ATP levels. **(A)** Live imaging of migratory wildtype, *Drp1 RNAi*, *Mfn OE*, and *Drp1 OE* border cells that also express *UAS-Lifeact-GFP*, which labels F-actin and outlines the leading protrusion. See Supplementary Videos S1–4 for details. **(B)** The protrusion length is measured as the distance between the tip of the protrusion to the periphery of the cluster. *Drp1 RNAi*, *Mfn OE*, and *Drp1 OE* border cell clusters have significantly shorter leading protrusions compared with the wildtype control. Each data point represents the length of the longest leading protrusion measured from each live imaging video, “n” is the number of videos examined for each genotype. A z-series of 9-10 confocal sections were taken every minute. After going through all the z-series, the confocal section with the longest protrusion visible was selected and measured. **(C)** Images of follicle cells stained with ATP5A (for mitochondria), phalloidin (for F-actin) and DAPI (for nuclei) with indicated genotypes. **(D)** Ovaries that express *Drp1 RNAi*, *Mfn OE*, and *Drp1 OE* by *Gr1-Gal4* have lower ATP levels compared with the control (*Gr1-Gal4* and *UAS-lacZ*). The relative ATP levels were normalized against the control (see Methods). **(E,F)** S2 cells transfected with *Drp1-HA* and *Mfn-HA* for 36, 48 and 60 h respectively. Their expression levels were analyzed by Western blot **(E)**, and their ATP levels were determined by ATP assay kits [**(F)**, see Methods]. **(G,H)** S2 cells transfected with *dsGFP* or *dsDrp1* for 36, 48 and 60 h respectively. Their expression levels were analyzed by Western blot **(G)**. The ATP levels of *dsDrp1* treated cells were significantly decreased from 36 to 60 h, as compare with the *dsGFP* control. Scale bars: 10 μm. Error bars indicate SD *p < 0.05, **p < 0.01, ***p < 0.001, ****p < 0.0001.

We next investigated the underlying cause of migration defects by *Drp1 RNAi*, *Mfn* or *Drp1 OE*, which supposedly caused disruption of mitochondrial dynamics. We used Lifeact-GFP to label the F-actin and actin cytoskeleton during live imaging of border cell migration. The wild-type border cell cluster utilizes a long leading protrusion enriched with F-actin at the leading edge of the cluster to power their collective migration (Figure 2A; Supplementary Video S1), reducing protrusion formation would lead to strong migration defects. *Drp1* knock down, *Mfn OE*, and *Drp1 OE* each led to shortened leading protrusions, as shown by live imaging of Lifeact-GFP (Figures 2A,B; Supplementary Videos S1–S4), and similar results was obtained by phalloidin staining of fixed samples (Supplementary Figure S5). Results from loss-of-function experiments indicate that Drp1-mediated mitochondrial fission is required for formation of long leading protrusion. On the other hand, gain-of-function Mfn and Drp1 results suggest that over-activation of fusion or fission also impairs protrusion formation. Formation of lamellipodial protrusion depends heavily on actin polymerization, an ATP-dependent process. We then sought to determine whether Drp1-mediated mitochondrial dynamics promote protrusion formation through regulating ATP levels during border cell migration. The small number of border cells for each egg chamber precludes direct ATP measurement in border cells. Instead, we proceeded to measure the ATP levels of ovaries that contain egg chambers at the early and middle stages of oogenesis. Each mid-stage egg chamber is composed of the somatic tissue of 650 follicle cells and the germline tissue of 15 nurse cells and one oocyte (Montell, 2003). Since border cells originate from the epithelial layer of follicle cells, results from follicle cells would closely reflect what would happen in border cells. Therefore, we drove the expression of *Drp RNAi*, *Drp1* and *Mfn* specifically in the follicle cells using the *Gr1-Gal4* driver, and found that loss-of-function of Drp1 or gain-of-function of Mfn resulted in increased mitochondrial fusion as compared to the control (Figure 2C). The *Mfn OE* or *Drp1 RNAi* follicle cells contains large patches of bright Mito-GFP signals (with *Mfn OE* more severe than *Drp1 RNAi*) that reflect a highly clustered mitochondrial morphology and are often asymmetrically distributed or concentrated in a corner of individual follicle cell (i.e., on one side of nucleus), whereas the control or *Drp1 OE* follicle cells exhibit a uniform distribution pattern of mitochondria around the central nucleus, likely due to their small size. Together, these results are similar to what we have observed in the border cells (Figures 1D, 2C), validating the use of follicles cells and thus egg chambers for ATP measurement.

We found that ATP levels were significantly decreased in *Drp1 RNAi* and *Mfn OE* as compared to that of the control (Figure 2D). It should be noted that if we can measure the ATP levels of only the follicle cells and not the nurse cells and oocyte (where Drp1 and Mfn levels are not altered), the extent of ATP level decrease would have been even greater than what we have observed. This result indicates that misregulation of mitochondrial fission results in decrease of ATP levels *in vivo*. To confirm this result, we utilized the S2 cells as an *in vitro* system to test the effects on ATP levels by misregulation of mitochondrial dynamics. *dsDrp1* as well as *Drp1* and *Mfn* were expressed in S2 cells to achieve *Drp1* knock down, *Drp1 OE*, and *Mfn OE* respectively. We found that *Drp1* loss-of-function and *Mfn* gain-of-function each resulted in significant increase of mitochondrial fusion accompanied by significant reduction of ATP levels (Figures 2E–H; Supplementary Figures S1, S2), similar to those observed in the follicle cells. This result indicates that Drp1-mediated mitochondrial fission is required for optimal ATP levels both *in vivo* and *in vitro*. Furthermore, overactivation of fission by *Drp1 OE* was shown to significantly increase mitochondrial fission or reduce mitochondria size (Supplementary Figure S1). Interestingly, *Drp1 OE* led to moderate increase of ATP levels 48 h after transfection but subsequent decrease of ATP levels 60 h after transfection. This result implies that moderate increase of Drp1 levels (at 48 h, Figure 2F), or moderate increase of fission is beneficial for ATP production. In contrast, strong increase of Drp1 levels (at 60 h, Figure 2F), or too much increase of fission is detrimental for ATP production. Taken together, the above results indicate that the reduced leading protrusions correlates with the reduction of ATP levels as resulted from the misregulation of mitochondrial dynamics, suggesting that during border cell migration Drp1 promotes formation of long leading protrusion through regulating mitochondrial fission and optimal ATP production.

### Reduction of ATP Levels Affects Actin and Myosin II Dynamics in Border Cells

**FIGURE 3:**
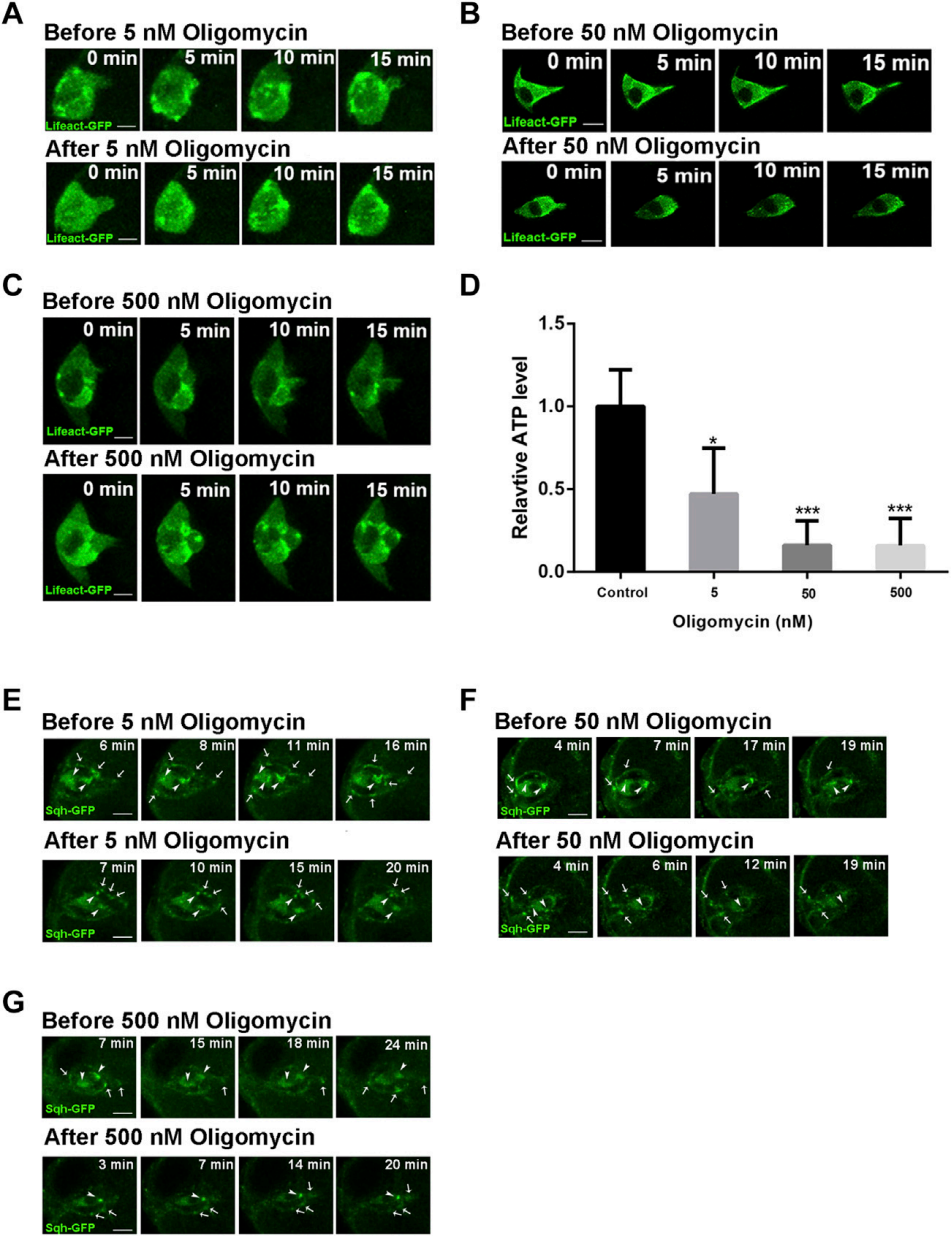
Actin and myosin dynamics are affected by ATP level reduction during border cell migration. **(A–C)** Before treatment of early stage nine egg chambers by 5, 50, and 500 nM Oligomycin respectively, the border clusters undergo dynamic cell shape changes, extend long leading protrusion, and exhibit strong dynamics of F-actin enriched patches. Less than 5 min after Oligomycin treatment, border cell clusters retract the leading protrusion and shape change and dynamic movement of F-actin patches were much reduced, with the effects by 50 and 500 nM treatment **(B,C)** more severe than that by 5 nM treatment **(A)**. See Supplementary Videos S5–10 for more details. **(D)** The ATP levels of ovaries were significantly decreased after treatment of 5, 50 and 500 nM Oligomycin as compared with the control (treated with DMSO). **(E–G)** Before treatment of early stage nine egg chambers by 5, 50, and 500 nM Oligomycin, border cells exhibit dynamic movement of Sqh (Myosin II’s regulatory light chain)-GFP enriched dots or patches along the cluster periphery or within the cluster center (polar cells). White arrows point to dynamic Sqh-GFP dots or patches near the outer periphery or surface of border cell clusters, while white arrowheads point to Sqh-GFP dots or patches within the two central polar cells, which display slower dynamic movement compared to those along the cluster periphery. After Oligomycin treatment, border cell clusters exhibit slower dynamic movement of Sqh-GFP patches or dots, with the effects by 50 and 500 nM treatment **(E,F)** more dramatic than that by 5 nM treatment **(G)**. Supplemental Videos S11–16 provide more details for comparison between the dynamics of Sqh-GFP before drug treatment and those after drug treatment. Scale bars: 10 μm. Error bars indicate SD *p < 0.05, **p < 0.01, ***p < 0.001.

We next sought to investigate the effect of ATP reduction on protrusion behavior during border cell migration. We treated the stage 9 egg chambers with Oligomycin, a drug that blocks ATP generation from mitochondria by inhibiting the proton channel of ATP synthase F0 subunit (Symersky et al., 2012), at different concentrations. Oligomycin treatment at 5 nM resulted in significant reduction of ATP levels in the ovaries, while higher concentrations of 50 and 500 nM resulted in strong reduction of ATP levels, indicating that the drug treatment is effective (Figure 3D). Live imaging revealed that treatment of 5 nM Oligomycin causes border cell cluster to retract leading protrusion immediately after drug treatment (Figure 3A; Supplementary Videos S5, S6). This phenotype is similar to what was observed for the *Drp1 RNAi* border cell cluster, which mostly exhibits a short leading protrusion and occasionally extends a long protrusion that was then quickly retracted (Supplementary Video S2). Treatment of 50 and 500 nM Oligomycin both resulted in similar retraction of long protrusions (Figures 3B,C; Supplementary Videos S7–S10). Furthermore, the highly dynamic F-actin patches labeled by Lifeact-GFP in the border cells before drug treatment exhibited significant reduction of dynamics after treatment, which is more severe in 50 and 500 nM treatment than that in 5 nM treatment. This dramatic reduction of dynamics of F-actin patches was not apparent in *Drp1 RNAi* border cells, likely due to the fact that drug treatment causes an immediate and drastic reduction in ATP levels whereas the effect on ATP levels from the genetic knockdown is over a longer time course and is less severe. We have recently found that a dynamic supracellular actomyosin network spanning throughout the outer surface of border cell cluster promotes a front polarized cluster morphology and collective migration (Wang et al., 2020). We therefore examined the effect of ATP reduction on Sqh (Myosin II’s regulatory light chain) organization and dynamics. Live imaging revealed that the assembly of Sqh-GFP into large patches as well as their dynamic movement on both the cluster surface and within polar cells (cluster interior) are significantly affected, with the 50 and 500 nM treatments being more severe than the 5 nM treatment (Figures 3E–G; Supplementary Videos S11–S16). Taken together, these results indicate that strong ATP reduction by Oligomycin treatment affects actin and myosin organization and dynamics, suggesting that reduction of ATP levels by *Drp1 RNAi* may result in similar (albeit milder) effects in border cells, negatively affecting protrusion behaviors.

### RhoA Induces Mitochondrial Fission Through Rho Kinase

**FIGURE 4:**
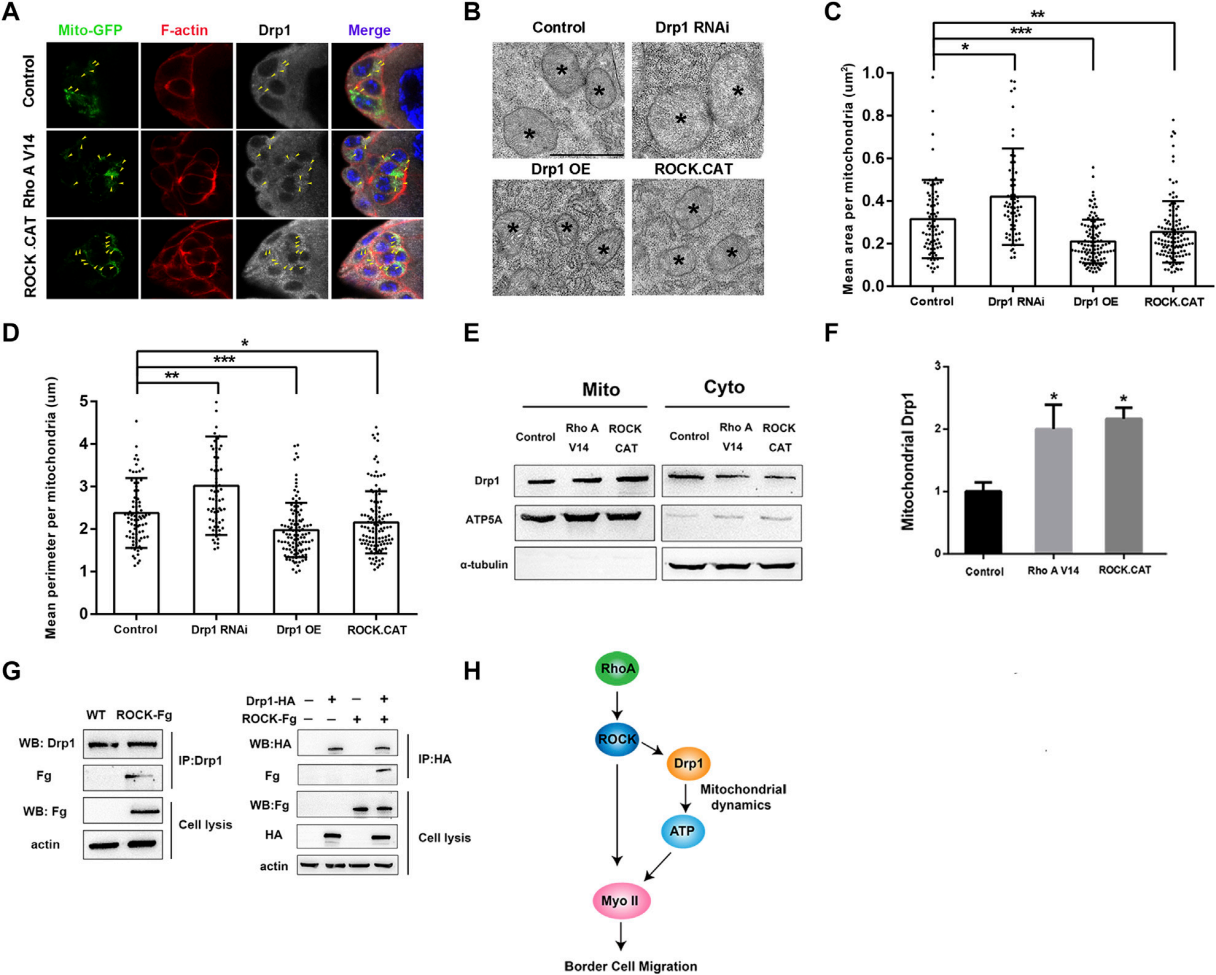
RhoA/ROCK signaling induces mitochondrial fission through interacting with Drp1. **(A)** Confocal images of early stage 9 border cell clusters stained with Mito-GFP (for mitochondria), phalloidin (for F-actin), Drp1 antibody and DAPI (for nuclei) with indicated genotypes. The colocalization of mitochondria and Drp1 is indicated by the yellow arrowhead. Expression of *RhoA V14* or *ROCK.CAT* leads to defects of rounded border cells and migration delay, as has previously been described (Bastock and Strutt, 2007), and the cell rounding is likely due to excessive contractile activity in the cortical actomyosin network as resulted from overactivation of Myosin II. **(B–D)** TEM analysis was performed for the follicle cells of stage 9 or 10 egg chambers with indicated genotypes. Mean area **(C)** and mean perimeter **(D)** per mitochondria were calculated and used as two indicators of mitochondrial size and morphology. Mean area and mean perimeter were measured by Image J. The mitochondria are from different egg chambers with indicated genotypes (*n* = 81, 63, 109 and 119 from left to right), respectively. However, due to the small size and the difficulty to locate border cell cluster within egg chambers, we were unable to obtain TEM images of the border cells. **(E,F)** Expressing *RhoA V14* or *ROCK.CAT* in the follicle cells by the *GR1-Gal4* driver increased levels of mitochondria-associated Drp1 in the ovaries. The whole proteins from ovaries were analyzed by Western blot with ATP5A and α-tubulin as mitochondrial and cytosolic markers respectively. **(G)** co-immunoprecipitation (co-IP) was performed using the newly generated antibody against *Drosophila* Drp1 (see Supplementary Figure S7 for methods and details). The endogenous Drp1 could be co-immunoprecipitated together with the exogenous ROCK-Fg, indicating that Drp1 binds to ROCK-Fg in S2 cells. Western blot of immunoprecipitates of Drp1-HA from S2 cells indicates that exogenously expressed Drp1-HA also physically interacts with ROCK-Fg in S2 cells. **(H)** A model showing Rho A/ROCK signaling in migratory border cells promoting Myosin II activity and dynamics by simultaneously regulating Drp1-mediated mitochondrial fission and direct phosphorylation of Myosin II (see Discussion for details). Scale bars: 10 μm in **(A)**, 1 μm in **(B)**. Error bars indicate SD *p < 0.05, **p < 0.01, ***p < 0.001, n.s., not significant.

RhoA and its downstream effector Rho kinase (ROCK) play critical roles in regulation of actin and Myosin II dynamics during border cell migration (Zhang et al., 2011; Montell et al., 2012; Wang et al., 2020). Recent studies in mammalian cells and animal models have also revealed that ROCK could directly phosphorylate and interact with Drp1 to either help recruit Drp1 to mitochondria to promote fission or prevent Drp1 from binding to mitochondria to suppress fission (Wang et al., 2012; Hu et al., 2020). We then sought to determine whether *Drosophila* RhoA and ROCK can regulate mitochondrial fission through affecting Drp1’s mitochondria binding ability. We overexpressed RhoA and ROCK or their activated forms in the S2 cells, follicle cells and border cells to test whether signaling from Rho A and ROCK affects Drp1’s recruitment to the mitochondria *in vitro* and *in vivo*. First, we found that overexpressing RhoA and ROCK in S2 cells did not alter the total levels of Drp1 level as compared to the control, suggesting that signaling from Rho A or ROCK does not change Drp1’s expression levels (Supplementary Figures S4A,B). Interestingly, RhoA and ROCK expression in S2 cells each caused significantly increased recruitment of Drp1 to the mitochondria, and treatment of ROCK inhibitor Y27632 abolished Rho’s positive effect on Drp1 recruitment, indicating that Rho A promotes Drp1 recruitment to the mitochondria through ROCK (Supplementary Figures S4C,D). Furthermore, Rho A and ROCK expression in S2 cells each resulted in significantly increased mitochondrial fission (Supplementary Figures S3A–D), as indicated by the significant reduction of four parameters of mitochondrial morphology. And treatment of ROCK inhibitor Y27632 also abolished Rho’s effect on increased fission, indicating that RhoA promotes mitochondrial fission through ROCK (Supplementary Figures S3A–D). Importantly, we also found that ROCK was able to physically interact with Drp1 (Figure 4G). We generated an antibody against *Drosophila* Drp1 and performed co-IP (Co-immunoprecipitation) assay using the newly generated Drp1 antibody. We found that the endogenous Drp1 in the S2 cells could be co-immunoprecipitated together with ROCK-Fg, and conversely ROCK-Fg could be co-immunoprecipitated together with Drp1-HA (Figure 4G). This result provides the mechanistic detail that ROCK regulates Drp1’s mitochondrial recruitment by binding to Drp1. Moreover, the effect of increased mitochondrial fission as a result of ROCK transfection was abolished by *Drp1* knockdown (dsDrp1) (Supplementary Figure S3E–H), indicating that Drp1 is required for ROCK-induced mitochondrial fission in S2 cells. Together, these results demonstrate that Rho signaling promotes Drp1’s mitochondrial recruitment and mitochondrial fission in S2 cells through ROCK and its physical interaction with Drp1.

Second, we found that expression of RhoA V14 and ROCK.CAT, the activated forms of RhoA and ROCK respectively, in the follicle cells each resulted in significant increase of Drp1 recruitment onto mitochondria without affecting Drp1’s total levels (Figures 4E,F; Supplementary Figures S4E,F), consistent with the *in vitro* results. Furthermore, TEM (transmission electron microscopy) analysis on stage 9 or 10 egg chambers confirmed that *ROCK.CAT* and *Drp1* expressing follicle cells contain significantly smaller mitochondria than the wildtype follicle cells, whereas *Drp1 RNAi* expressing cells display significantly larger mitochondria (Figures 4B–D). Therefore, both *in vivo* and *in vitro* results support the conclusion that RhoA signaling induces mitochondrial fission through ROCK. Interestingly, expression of *RhoA V14* or *ROCK.CAT* in the follicle cells results in higher ATP levels in the egg chambers as compared with the control (Supplementary Figure S6), further supporting the notion that increased mitochondrial fission (as caused by elevated Rho or ROCK activity) promotes higher ATP levels. Lastly, confocal microscopy demonstrates that increasing ROCK or Rho activity in the border cells (by *ROCK.CAT* or *RhoA V14* expression) could increase the staining of Drp1 that colocalizes with mitochondria as compared with that of the wildtype control (Figure 4A). This result is consistent with the data that Rho and ROCK increase the recruitment of Drp1 to the mitochondria in the follicle cells (Figures 4E,F) and S2 cells (Supplementary Figures S4A–D).

## Discussion

In this study, we show that *Drp1* but not *Mfn* is genetically required for border cell migration, suggesting that Drp1-mediated fission plays critical roles in collective cell migration. Since fission and fusion oppose each other and form an equilibrium for the mitochondrial dynamics, lack of Drp1-mediated fission means unopposed fusion. Indeed, excessive fusion as resulted by Mfn overexpression also disrupted border cell migration, consistent with the migration defects caused by Drp1 loss-of-function. One reason that Drp1-mediated fission is critical for border cell migration is that a more fragmented mitochondrial network may be more easily remodeled and more uniformly distributed to suit the metabolic needs of migratory border cells and that an overly fused mitochondrial network is more rigid and less likely to be remodeled. Consistent with this notion, we found that Mfn deficiency, which leads to unopposed fission, did not result in significant migration defects. Interestingly, a number of mammalian studies have previously shown that Drp1-mediated mitochondrial fission promotes single cell migration in various cell types, including T cells, breast cancer cells, thyroid cancer cells, and neural progenitor cells (Campello et al., 2006; Zhao et al., 2013; Ferreira-da-Silva et al., 2015; Kim et al., 2015; Simula et al., 2018). Specifically, chemotaxis of lymphocytes and metastasis of breast cancer cells had demonstrated that mitochondrial fission was beneficial for migration of both lymphocytes and breast cancer cells but too much fusion suppressed their migration (Campello et al., 2006; Zhao et al., 2013). In addition, both studies have shown a positive correlation of ATP levels and migratory ability, suggesting that mitochondrial fission promotes optimal ATP levels (Campello et al., 2006; Zhao et al., 2013). Moreover, both Drp1 deficiency and Mfn1 overexpression in breast cancer cells inhibited formation of lamellipodial formation, which is essential for metastasis (Zhao et al., 2013). Consistently, we found that both Drp1 deficiency and Mfn1 overexpression in the border cells caused significant reduction in the length of leading protrusions, which are required for the collective migration of border cells. And such defects in leading protrusions could be due to reduction in the ATP levels, since Drp1 deficiency or Mfn1 overexpression could each decrease ATP levels *in vivo* (ovary) and *in vitro* (S2 cells). Indeed, sudden reduction of ATP levels by Oligomycin treatment caused immediate retraction of leading protrusions. Together, these results suggest that unopposed or excessively fused mitochondrial networks are not favorable for optimal ATP production during border cell migration.

Drp1-mediated mitochondrial fission may also be important for promoting actomyosin dynamics in the border cells through its regulation of ATP levels. Our recent work has demonstrated that a dynamic supracellular actomyosin network spanning across the whole border cell cluster mediates cell-cell communication between adjacent border cells, resulting in the cluster’s front-polarized morphology and efficient migration (Wang et al., 2020). Myosin II patches within the network were observed to undergo directional movement or flow along outer cortex of border cells, and they were thought to be important for protrusion formation and front-polarized morphology in the border cells (Wang et al., 2020). In this study, we show that reduction of ATP levels by Oligomycin reduced the dynamic movement or flow of these Myosin II patches as well as the F-actin enriched structures, suggesting that during border cell migration mitochondrial dynamics may promote actomyosin dynamics through ATP generation. Besides ATP generation, mitochondria are also known to regulate intracellular calcium levels. A previous study has shown that increasing mitochondrial fission in the axon promotes uptake of Ca^2+^ into the mitochondria, leading to reduced presynaptic cytoplasmic Ca^2+^ levels and subsequently decreased presynaptic release of neurotransmitters (Lewis et al., 2018). So, there remains a possibility that mitochondrial dynamics could somehow affect actomyosin dynamics through its regulation of cytoplasmic Ca^2+^, which is known to signal to and regulate the actomyosin network *via* calmodulin and MLCK, a kinase that phosphorylates and activates Myosin II (Somlyo and Somlyo, 2003).

Our data point to Drp1 as an attractive target for upstream signaling that are active during migration to regulate mitochondrial dynamics. Indeed, we find that RhoA/ROCK signaling, which is essential for actin and myosin dynamics during border cell migration, could exert its effect on mitochondria fission through regulating Drp1’s recruitment to mitochondria both *in vivo* and *in vitro* (Figure 4H). Since ROCK physically interacts with Drp1, one likely mode of regulation by the serine/threonine kinase ROCK could be ROCK directly phosphorylating Drp1. Mammalian studies had previously shown that mammalian Drp1 could be phosphorylated by ROCK1 at three different phosphorylation sites, resulting in either activation or inactivation of Drp1 depending on its physiological context (Wang et al., 2012; Hoppins, 2014; Hu et al., 2020). Whether ROCK phosphorylates and activates Drp1 in migratory border cells or in other *Drosophila* cell types remains to be elucidated. These findings support a general scenario in migratory cells, in which dynamics of both actomyosin network and mitochondrial network need to be simultaneously regulated and coordinated (Figure 4H). And our study reveals that RhoA and ROCK may couple or coordinate these two cellular processes to achieve optimal actin and myosin function, leading to protrusive and migratory behavior (Figure 4H).

## Data Availability

The original contributions presented in the study are included in the article/Supplementary Material, further inquiries can be directed to the corresponding authors.

## Supplementary Material

Supplementary Figure S1 Drp1 and Mfn regulate mitochondrial morphology in S2 cells. **(A)** Confocal images showing S2 cell transfected with Drp1-HA (green) and Mfn-HA (green). Mitochondria are labelled by Mitotracker. **(B and C)** Mean area **(B)** and mean perimeter **(C)** per mitochondria in each cell were calculated by the Image J plugin Mitochondria Analyzer and used as two indicators of mitochondrial size (see Methods). Mitochondria in Drp1-HA transfected S2 cells (n = 25) are significantly decreased in both mean area **(B)** and mean perimeter **(C)** as compared to the control (n = 25). Mitochondria in Mfn-HA transfected S2 cells (n = 25) are significantly increased in both mean area **(B)** and mean perimeter **(C)** as compared to the control. **(D)** Form factor and aspect ratio were calculated by Mitochondria Analyzer as two indicators of mitochondrial shape to assess circularity or roundness of mitochondria. Both form factor and aspect ratio are decreased in Drp1-HA transfected cells (n = 25), indicating a more rounded shape in mitochondria, and are increased in Mfn-HA (n = 25) transfected cells, indicating a less rounded or more elongated shape of mitochondria. In **(B–D)**, n indicates the number of confocal images or number of individual cells (each image contains one S2 cell) used for analysis of mean area, mean perimeter, form factor and aspect ratio by the Mitochondria Analyzer. Scale bars: 5 μm. Error bars indicate S.D.*p < 0.05, **p < 0.01, ***p < 0.001.

Supplementary Figure S2 Drp1 knock down induces mitochondrial fusion in S2 cells. **(A)** Confocal images showing S2 cell transfected with dsGFP (control) or dsDrp1. **(B–D)** Mitochondria in dsDrp1 transfected S2 cells (n = 20) are significantly increased in both mean area **(B)** and mean perimeter **(C)** as compared to the control, indicating significant increase of mitochondrial size. **(D)** Both form factor and aspect ratio are not significantly increased in dsDrp1 transfected S2 cells (n = 20), indicating no significant change of mitochondrial shape or roundness. Scale bars: 5 μm. Error bars indicate S.D. *p < 0.05, **p < 0.01. n.s., not significant.

Supplementary Figure S3
**(A)** Confocal images showing S2 cell transfected or treated with RhoA-HA, ROCK-HA and ROCK inhibitor Y-27632 (10 μM). Mitochondria are labelled by Mitotracker. **(B–D)** From the confocal images, mean area **(B)** and mean perimeter **(C)** per mitochondria were calculated and used as two indicators of mitochondrial size. Mitochondria in RhoA and ROCK transfected S2 cells (n = 20) are significantly decreased in both mean area **(B)** and mean perimeter **(C)** as compared to the control. Mitochondria in S2 cells treated with ROCK inhibitor Y-27632 (10 μM) are not significantly decreased in both mean area **(B)** and mean perimeter **(C)** as compared to the control. **(D)** Form factor and aspect ratio, the two indicators of mitochondrial shape or roundness, are significantly decreased in Rho A and ROCK transfected cells (n = 20). **(E–H)** From the confocal images **(E)**, mean area, mean perimeter **(F)**, form factor and aspect ratio **(G)** were calculated to assess mitochondrial size and shape. Mitochondria in ROCK-HA+dsDrp1 co-transfected S2 cells are significantly increased in both mean area and mean perimeter **(F)** as compared to the control. **(G)** Form factor is significantly increased in S2 cells transfected with ROCK-HA+dsDrp1 as compared to the control, whereas aspect ratio shows no significant difference. **(H)** Western blot analysis shows that the Drp1 level in S2 cells transfected with ROCK-HA+dsDrp1 decreased as compared to the ROCK-HA+dsGFP, indicating efficient Drp1 knock down. Scale bars: 5 μm. Error bars indicate S.D. *p < 0.05, **p < 0.01, ***p < 0.001, n.s., not significant.

Supplementary Figure S4
**(A and B)** Western blot analysis of S2 cells transfected or treated with RhoA-HA, ROCK-HA, and ROCK plus inhibitor Y-27632 (10 μM). The total protein levels of Drp1 are not affected by transfection with RhoA-HA, ROCK-HA, or ROCK-HA+ Y-27632. **(C and D)** The levels of mitochondrial Drp1 were increased after transfection of Rho A-HA or ROCK-HA into the S2 cells. The increase was abolished after adding ROCK inhibitor Y-27632 to the Rho A-HA transfected S2 cells. Protein level quantification and statistical analysis were done for experiments that were repeated for three times. **(E and F)** Expressing RhoA V14 or ROCK.CAT in the follicle cells by the Gr1-Gal4 driver did not affect the total protein levels of Drp1 in the ovaries. The whole proteins from ovaries were analyzed by Western blot. Error bars indicate S.D. *p < 0.05, **p < 0.01, ***p < 0.001, n.s., not significant.

Supplementary Figure S5
**(A)** Confocal images showing border cell cluster expressing Drp1 RNAi, Mfn (Mfn OE) and Drp1 (Drp1 OE) by the slbo-Gal4. **(B)** The protrusion length is measured as the distance between the tip of the protrusion to the periphery of the cluster as previously described (Zhang et al, 2011), actin-enriched protrusion is highlighted by rhodamine-phalloidin, which stains F-actin (red) in the fixed samples. DAPI stains the nuclei (blue). Drp1 RNAi, Mfn OE and Drp1 OE border cell clusters have significantly shorter leading protrusions than the wildtype control. Scale bars: 10 mm. Error bars indicate S.D. **p < 0.01, ***p < 0.001. n.s., not significant.

Supplementary Figure S6
**(A)** Confocal images showing follicle cells expressing RhoA V14 and ROCK.CAT by the GR1-Gal4. An increase of F-actin in the cell cortex of follicle cells was observed as compared with the control. **(B)** Significant increase of ATP levels was also detected in egg chambers with RhoA V14 and ROCK.CAT expression by the GR1-Gal4. Scale bars: 10 mm. Error bars indicate S.D. **p < 0.01. n.s., not significant.

Supplementary Figure S7 Generation of the Drp1 antibody. **(A)** Diagram of the fragment of Drp1 that was used to generate polyclonal antibody. **(B)** Lane M: protein marker; Lane 1: negative control for rDrp1 (without IPTG induction); Lane 2: IPTG induced recombinant Drp1 (rDrp1); Lane 3: purified rDrp1. **(C)** The specific antibody detection of endogenous Drp1 from S2 cells. **(D)** Expressing UAS-Drp1 RNAi and UAS-LacZ (control) in the follicle cells by the Gr1-Gal4 driver. The whole proteins from ovaries were analyzed by SDS-PAGE and Western blot. The preparation of polyclonal antibody was carried out as previously described (Qu et al., 2018). The sequences of Drp1 were amplified with specific primers (rDrp1-His-F and -R) (Supplementary Table S1). The PCR products were inserted into pET-32a vector with a His-tag. The recombinant plasmid was used to transform E. coli BL21 (DE3) competent cells. The proteins were purified by a Ni^2+^ chelating sepharose column, and their concentrations were measured by Bradford method. The recombinant protein was used to immunize 6-weeks old mice to acquire polyclonal antibody.

Supplementary Table S1 Primers used in this study.
