# Supplementary material for: RhoA/ROCK Signaling Regulates Drp1-Mediated Mitochondrial Fission During Collective Cell Migration
Source: Front Cell Dev Biol. 2022 May 31;10:882581. doi: 10.3389/fcell.2022.882581 (PMC9194559; doi:10.3389/fcell.2022.882581)
Supplement: Supplementary file 2 [file DataSheet2.docx]

**Supplementary Figures and Table**


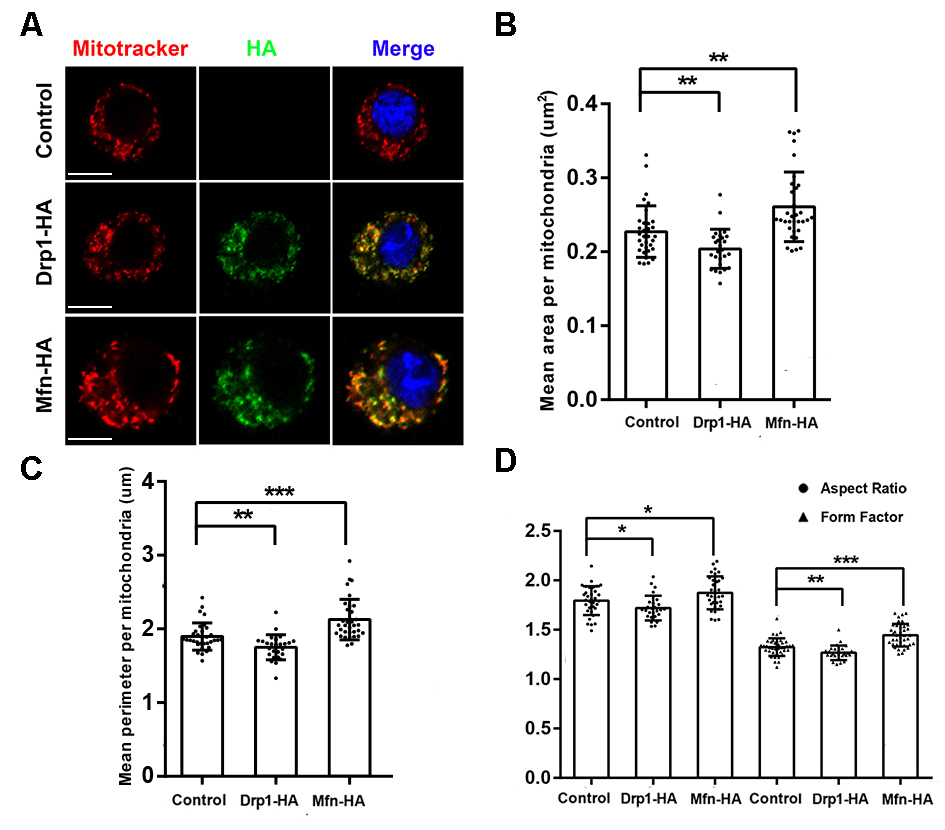


**Supplementary Figure 1. Drp1 and Mfn regulate mitochondrial morphology in S2 cells.**

(A) Confocal images showing S2 cell transfected with Drp1-HA (green) and Mfn-HA (green). Mitochondria are labelled by Mitotracker. (B and C) Mean area (B) and mean perimeter (C) per mitochondria in each cell were calculated by the Image J plugin Mitochondria Analyzer and used as two indicators of mitochondrial size (see Methods). Mitochondria in Drp1-HA transfected S2 cells (n=25) are significantly decreased in both mean area (B) and mean perimeter (C) as compared to the control (n=25). Mitochondria in Mfn-HA transfected S2 cells (n=25) are significantly increased in both mean area (B) and mean perimeter (C) as compared to the control. (D) Form factor and aspect ratio were calculated by Mitochondria Analyzer as two indicators of mitochondrial shape to assess circularity or roundness of mitochondria. Both form factor and aspect ratio are decreased in Drp1-HA transfected cells (n=25), indicating a more rounded shape in mitochondria, and are increased in Mfn-HA (n=25) transfected cells, indicating a less rounded or more elongated shape of mitochondria. In B-D, n indicates the number of confocal images or number of individual cells (each image contains one S2 cell) used for analysis of mean area, mean perimeter, form factor and aspect ratio by the Mitochondria Analyzer. Scale bars: 5 μm. Error bars indicate S.D.*P<0.05, **P<0.01, ***P<0.001.


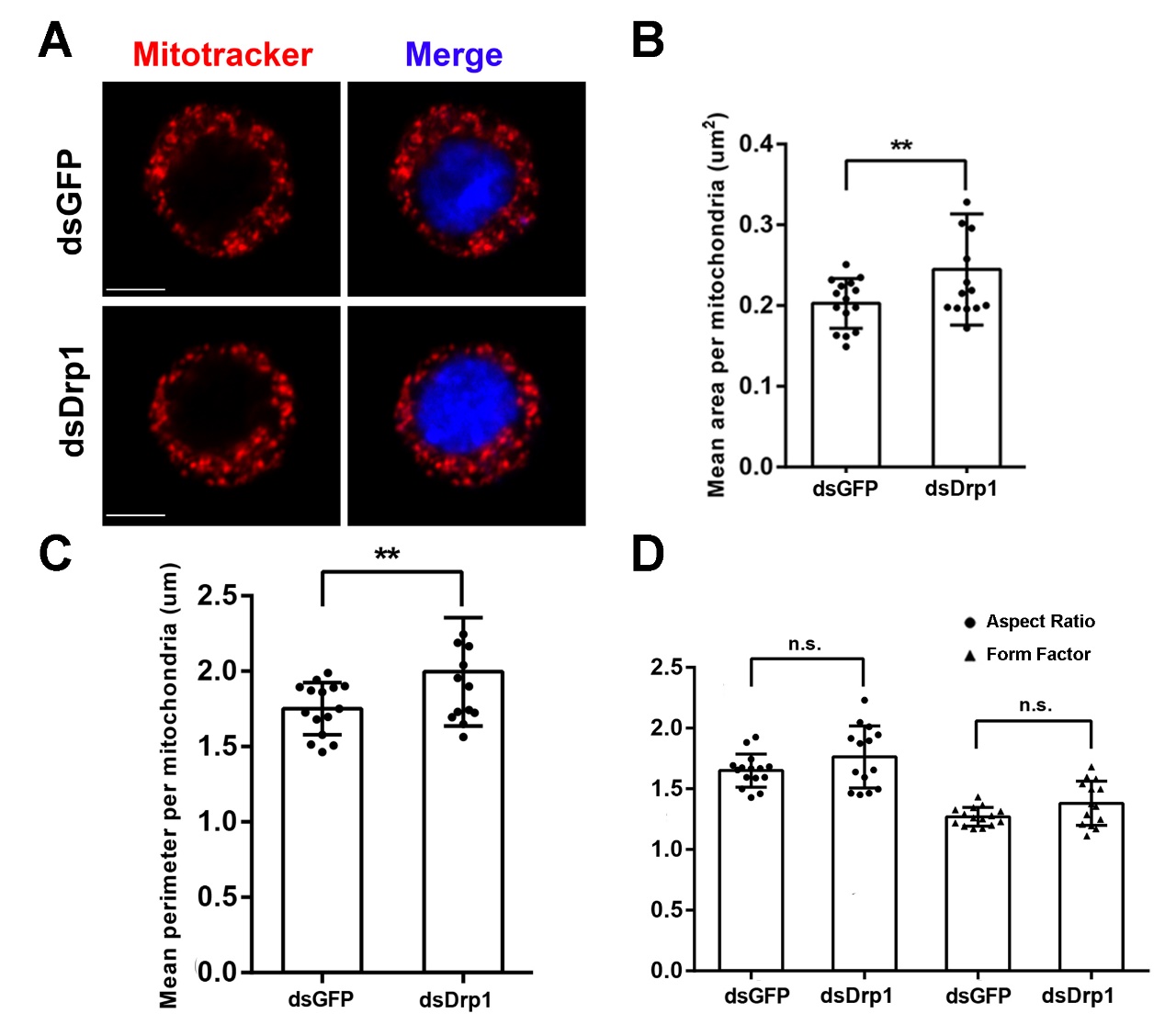


**Supplementary Figure 2. Drp1 knock down induces mitochondrial fusion in S2 cells.**

(A) Confocal images showing S2 cell transfected with dsGFP (control) or dsDrp1. (B-D) Mitochondria in dsDrp1 transfected S2 cells (n=20) are significantly increased in both mean area (B) and mean perimeter (C) as compared to the control, indicating significant increase of mitochondrial size. (D) Both form factor and aspect ratio are not significantly increased in dsDrp1 transfected S2 cells (n=20), indicating no significant change of mitochondrial shape or roundness. Scale bars: 5 μm. Error bars indicate S.D. *P<0.05, **P<0.01. n.s., not significant.


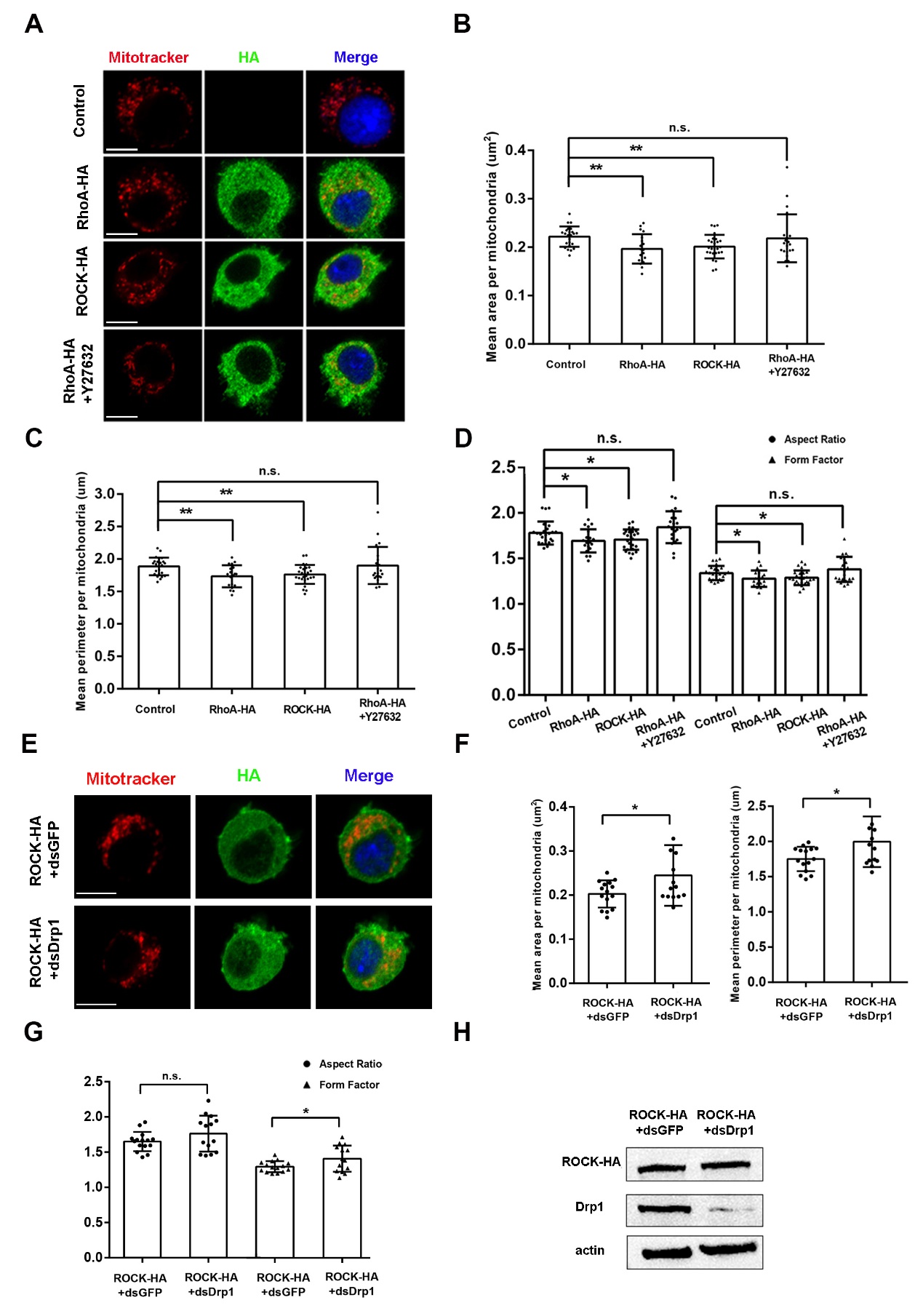


**Supplementary Figure 3.**

(A) Confocal images showing S2 cell transfected or treated with RhoA-HA, ROCK-HA and ROCK inhibitor Y-27632 (10 μM). Mitochondria are labelled by Mitotracker. (B-D) From the confocal images, mean area (B) and mean perimeter (C) per mitochondria were calculated and used as two indicators of mitochondrial size. Mitochondria in RhoA and ROCK transfected S2 cells (n=20) are significantly decreased in both mean area (B) and mean perimeter (C) as compared to the control. Mitochondria in S2 cells treated with ROCK inhibitor Y-27632 (10 μM) are not significantly decreased in both mean area (B) and mean perimeter (C) as compared to the control. (D) Form factor and aspect ratio, the two indicators of mitochondrial shape or roundness, are significantly decreased in Rho A and ROCK transfected cells (n=20). (E-H) From the confocal images (E), mean area, mean perimeter (F), form factor and aspect ratio (G) were calculated to assess mitochondrial size and shape. Mitochondria in ROCK-HA＋dsDrp1 co-transfected S2 cells are significantly increased in both mean area and mean perimeter (F) as compared to the control. (G) Form factor is significantly increased in S2 cells transfected with ROCK-HA＋dsDrp1 as compared to the control, whereas aspect ratio shows no significant difference. (H) Western blot analysis shows that the Drp1 level in S2 cells transfected with ROCK-HA+dsDrp1 decreased as compared to the ROCK-HA+dsGFP, indicating efficient Drp1 knock down. Scale bars: 5 μm. Error bars indicate S.D. *P<0.05, **P<0.01, ***P<0.001, n.s., not significant.

**
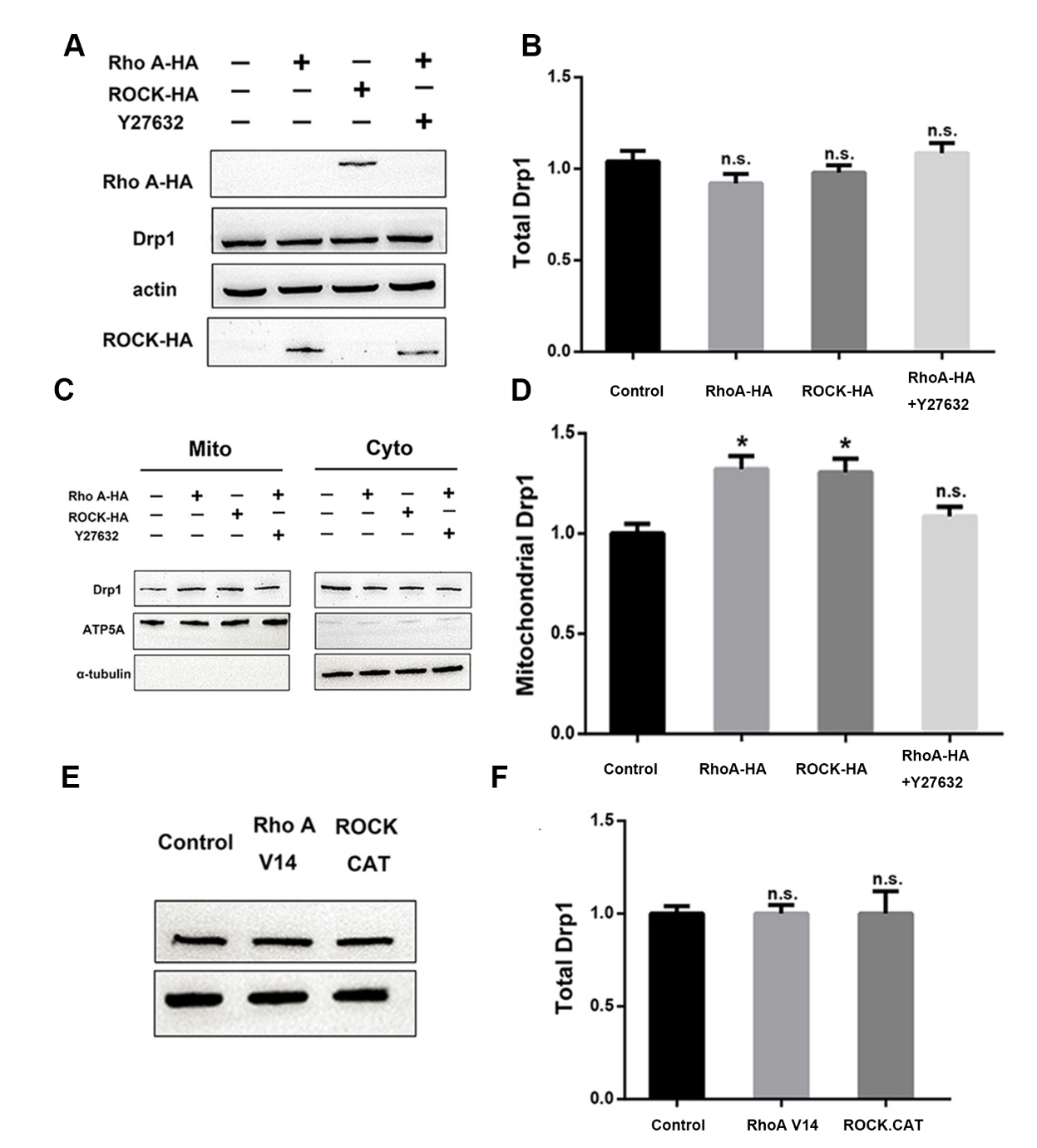
**

**Supplementary Figure 4.**

(A and B) Western blot analysis of S2 cells transfected or treated with RhoA-HA, ROCK-HA, and ROCK plus inhibitor Y-27632 (10 μM). The total protein levels of Drp1 are not affected by transfection with RhoA-HA, ROCK-HA, or ROCK-HA+ Y-27632. (C and D) The levels of mitochondrial Drp1 were increased after transfection of Rho A-HA or ROCK-HA into the S2 cells. The increase was abolished after adding ROCK inhibitor Y-27632 to the Rho A-HA transfected S2 cells. Protein level quantification and statistical analysis were done for experiments that were repeated for three times. (E and F) Expressing *RhoA V14* or *ROCK.CAT* in the follicle cells by the *Gr1-Gal4* driver did not affect the total protein levels of Drp1 in the ovaries. The whole proteins from ovaries were analyzed by Western blot. Error bars indicate S.D. *P<0.05, **P<0.01, ***P<0.001, n.s., not significant.


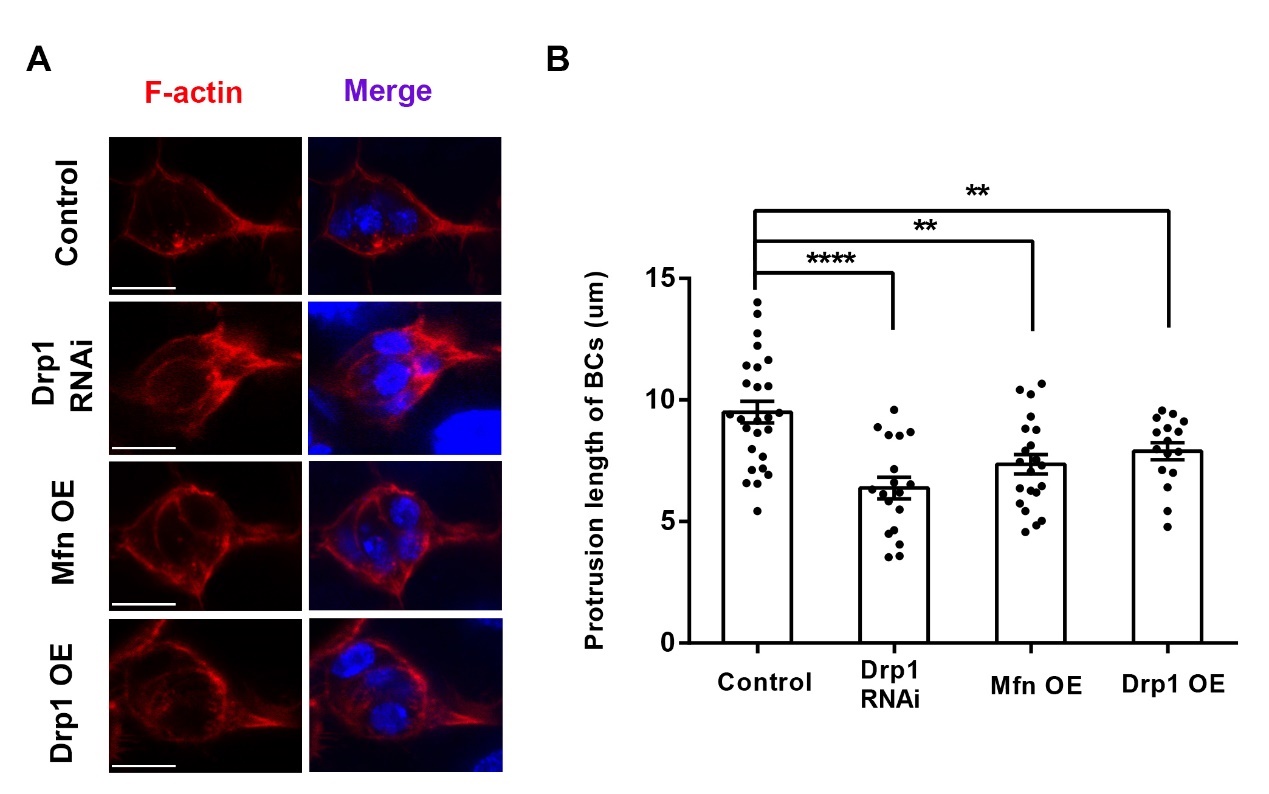


**Supplementary Figure 5.**

(A) Confocal images showing border cell cluster expressing *Drp1 RNAi*, *Mfn* *(Mfn OE)* and *Drp1 (Drp1 OE)* by the *slbo-Gal4*. (B) The protrusion length is measured as the distance between the tip of the protrusion to the periphery of the cluster as previously described (Zhang et al, 2011), actin-enriched protrusion is highlighted by rhodamine-phalloidin, which stains F-actin (red) in the fixed samples. DAPI stains the nuclei (blue). *Drp1 RNAi*, *Mfn OE* and *Drp1 OE* border cell clusters have significantly shorter leading protrusions than the wildtype control. Scale bars: 10 μm. Error bars indicate S.D. **P<0.01, ****P<0.0001, n.s., not significant.


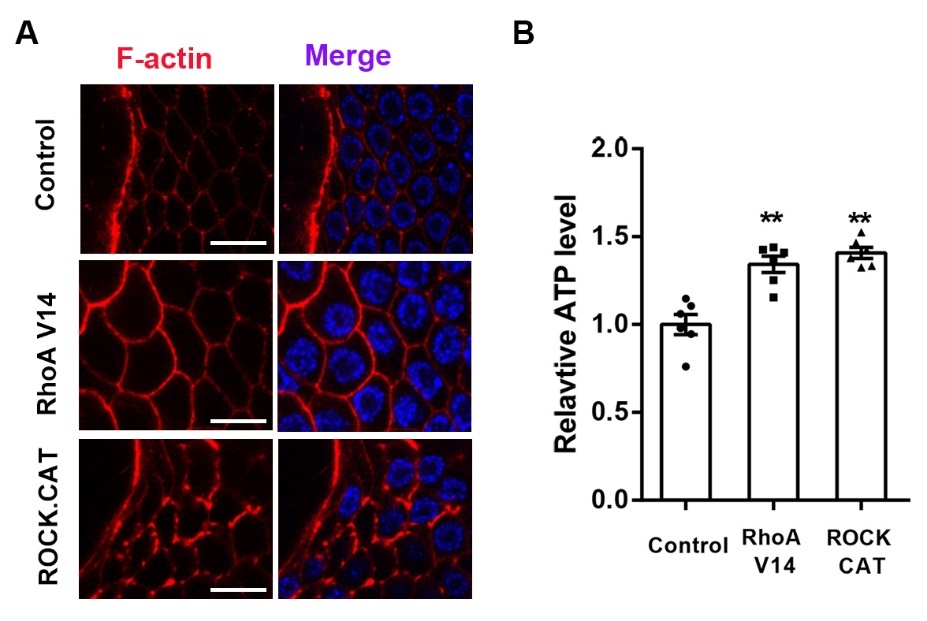


**Supplementary Figure 6.**

(A) Confocal images showing follicle cells expressing *RhoA V14* and *ROCK.CAT* by the *GR1-Gal4.* An increase of F-actin in the cell cortex of follicle cells was observed as compared with the control. (B) Significant increase of ATP levels was also detected in egg chambers with *RhoA V14* and *ROCK.CAT* expression by the *GR1-Gal4*. Scale bars: 10 μm. Error bars indicate S.D. **P<0.01, n.s., not significant.


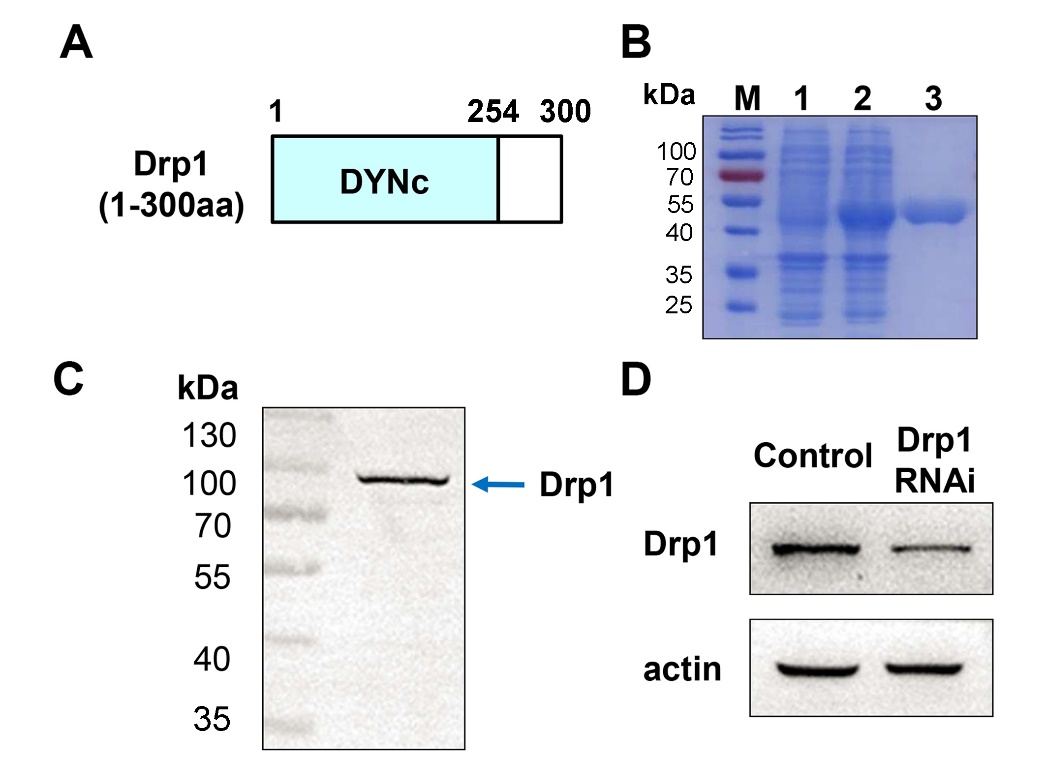


**Supplementary Figure 7. Generation of the Drp1 antibody.**

(A) Diagram of the fragment of Drp1 that was used to generate polyclonal antibody. (B) Lane M: protein marker; Lane 1: negative control for rDrp1 (without IPTG induction); Lane 2: IPTG induced recombinant Drp1 (rDrp1); Lane 3: purified rDrp1. (C) The specific antibody detection of endogenous Drp1 from S2 cells**.** (D) Expressing *UAS-Drp1 RNAi* and *UAS-LacZ* (control) in the follicle cells by the *Gr1-Gal4* driver. The whole proteins from ovaries were analyzed by SDS-PAGE and Western blot. The preparation of polyclonal antibody was carried out as previously described (Qu et al., 2018). The sequences of Drp1 were amplified with specific primers (rDrp1-His-F and -R) (Supplementary Table 1). The PCR products were inserted into pET-32a vector with a His-tag. The recombinant plasmid was used to transform *E. coli* BL21 (DE3) competent cells. The proteins were purified by a Ni^2+^ chelating sepharose column, and their concentrations were measured by Bradford method. The recombinant protein was used to immunize 6-weeks old mice to acquire polyclonal antibody.

**Supplementary Table 1. Primers used in this study.**

| **Oligonucleotide Name** | **Sequence** |
| --- | --- |
| Drp1-HA-F | TGAATACAAGAAGAGAACTCTGAATAGGGAATTGGATGGAGGCCCTAATTCCGGT |
| Drp1-HA-R | TAGCCCGCATAGTCAGGAACATCGTATGGGTACATCCACATGTGGGTCTCGCGGA |
| Mfn-HA-F | TGAATACAAGAAGAGAACTCTGAATAGGGAATTGGATGGCGGCCTACTTGAACCGCACC |
| Mfn-HA-R | TAGCCCGCATAGTCAGGAACATCGTATGGGTACATCTGCGGCGATATATAGTTGTGCTC |
| rDrp1-His-F | CTGCTGCTAAATTCGAACGCCAGCACATGGACAGCATGGAGGCCCTAATTCCGGT |
| rDrp1-His-R | TTAGCAGCCGGATCTCAGTGGTGGTGGTGGTGGTGCAGGCAGTCCCGGATGTGGT |
| ROCK-Fg-F | AACTCTGAATAGGGAATTGGGAATTCGTTAATGCCAGCTGGACGAGAAAC |
| ROCK-Fg-R | ATAATCACCGTCATGGTCTTTGTAGTCCATTTTCAGCGATGAATTGGCTG |
| Rho1-HA-F | TGAATACAAGAAGAGAACTCTGAATAGGGAATTGGATGACGACGATTCGCAAGAA |
| Rho1-HA-R | TAGCCCGCATAGTCAGGAACATCGTATGGGTACATGAGCAAAAGGCATCTGGTCT |
| ROCK-HA-F | TGAATACAAGAAGAGAACTCTGAATAGGGAATTGGATGCCAGCTGGACGAGAAAC |
| ROCK-HA-R | TAGCCCGCATAGTCAGGAACATCGTATGGGTACATCTTGTCATCGTCATCCTTGT |
| dsGFP-F | GAATTAATACGACTCACTATAGGGAGAATGGTGAGCAAGGGCGAGGAGCT |
| dsGFP-R | GAATTAATACGACTCACTATAGGGAGACTTGTACAGCTCGTCCATGCCGA |
| dsDRP1-F | GAATTAATACGACTCACTATAGGGAGAATGGAGGCCCTAATTCCGGTCATAA |
| dsDRP1-R | GAATTAATACGACTCACTATAGGGAGACAGGCAGTCCCGGATGTGGTGCATC |
